# Systematic pattern analyses of Vδ2^+^ TCRs reveal that shared “public” Vδ2^+^ γδ T cell clones are a consequence of rearrangement bias and a higher expansion status

**DOI:** 10.3389/fimmu.2022.960920

**Published:** 2022-09-27

**Authors:** Lihua Deng, Anna Harms, Sarina Ravens, Immo Prinz, Likai Tan

**Affiliations:** ^1^ Institute of Systems Immunology, University Medical Center Hamburg-Eppendorf, Hamburg, Germany; ^2^ Institute of Immunology, Hannover Medical School, Hannover, Germany

**Keywords:** γδ TCR, Vγ9Vδ2^+^ T cells, TCR distance, TCR sequencing, *TRD* rearrangement

## Abstract

**Background:**

Vγ9Vδ2^+^ T cells are a major innate T cell subset in human peripheral blood. Their Vδ2^+^ VDJ-rearrangements are short and simple in the fetal thymus and gradually increase in diversity and CDR3 length along with development. So-called “public” versions of Vδ2^+^ TCRs are shared among individuals of all ages. However, it is unclear whether such frequently occurring “public” Vγ9Vδ2^+^ T cell clones are derived from the fetal thymus and whether they are fitter to proliferate and persist than infrequent “private” clones.

**Methods:**

Shared “public” Vδ2^+^ TCRs were identified from Vδ2^+^ TCR-repertoires collected from 89 individuals, including newborns (cord blood), infants, and adults (peripheral blood). Distance matrices of Vδ2^+^ CDR3 were generated by TCRdist3 and then embedded into a UMAP for visualizing the heterogeneity of Vδ2^+^ TCRs.

**Results:**

Vδ2^+^ CDR3 distance matrix embedded by UMAP revealed that the heterogeneity of Vδ2^+^ TCRs is primarily determined by the J-usage and CDR3aa length, while age or publicity-specific motifs were not found. The most prevalent public Vδ2^+^ TCRs showed germline-like rearrangement with low N-insertions. Age-related features were also identified. Public Vδ2^+^ *TRDJ1* TCRs from cord blood showed higher N-insertions and longer CDR3 lengths. Synonymous codons resulting from VDJ rearrangement also contribute to the generation of public Vδ2^+^ TCRs. Each public TCR was always produced by multiple different transcripts, even with different D gene usage, and the publicity of Vδ2^+^ TCRs was positively associated with expansion status.

**Conclusion:**

To conclude, the heterogeneity of Vδ2^+^ TCRs is mainly determined by *TRDJ*-usage and the length of CDR3aa sequences. Public Vδ2^+^ TCRs result from germline-like rearrangement and synonymous codons, associated with a higher expansion status.

## Introduction

γδ T cells are unconventional T cells which have T cell receptors (TCR) consisting of both rearranged γ (TRG gene) and δ (TRD gene) chains. Like αβ T cells, γδ T cells use the recombination of variable, diversity, and joining gene segments (V(D)J recombination) to generate the complementarity-determining region 3 (CDR3) of the *TRG* and *TRD*. The diversity of these CDR3 regions is further amplified by the insertion of palindromic sequences (P nucleotides) and additional non-templated nucleotides (N-insertions) introduced by terminal deoxynucleotidyl transferase (TdT) (1, 2).

However, in contrast to conventional αβ T cells, which use numerous V segments almost randomly, human γδ T cells exclusively use Vδ1, Vδ2, and to a lesser extent also Vδ3 segments to generate delta chains. Further restrictions on diversity are imposed due to Vδ2^+^ chains mostly pairing with Vγ9-JP chains (2). The resulting Vγ9Vδ2^+^ T cells are regarded as innate γδ effectors that are quickly activated in anti-tumor, infection, and inflammation within diseases (3). Committed Vγ9Vδ2^+^ T effector cells are enriched in fetal thymus and blood, where they then persist into adulthood (4–6). The Vγ9Vδ2^+^ TCRs uniformly recognize phosphoantigens like microbial-derived (E)-4-hydroxy-3-methyl-but-2-enyl pyrophosphate (HMB-PP) and host-derived Isopentenyl pyrophosphate (IPP) in a pMHC-unrestricted manner (7–10), leading to fast TCR expansion and cytokine release of Vγ9Vδ2^+^ γδ T cells (3). The Vγ9Vδ2^+^ TCRs are featured as “semi-invariant” TCRs whereby the Vγ9 chains always have a *TRGV9*-*TRGJP* rearrangement. Fetal-derived Vγ9JP chains often express the germline-encoded CDR3 sequence CALWEVQELGKKIKVF due to the lack of TdT in the fetal thymus (5, 6). The Vδ2^+^ repertoire, on the other hand, that evolves during human development remains both highly diverse and individual (4, 5). In the early stages of life, the *TRDV2* gene segments preferentially rearrange with *TRDJ3* and *TRDJ2*, and gradually switch to *TRDJ1* after birth (11). Meanwhile, more N-insertions and longer CDR3 length are introduced into Vδ2^+^ TCRs after birth due to the increasing activity of the TdT (6). Public Vδ2^+^ TCRs are frequent among Vδ2^+^ repertoires from both the fetus and cord blood (6, 12–14). Public Vδ2^+^ TCRs have a higher overall diversity than the public Vγ9-JP; they occupy a substantial portion of Vδ2^+^ repertoires from adult peripheral blood (4). However, the properties and ontogeny of public Vγ9Vδ2^+^ TCRs are not completely solved. It is also unclear whether public Vγ9Vδ2^+^ TCRs have any advantage in target recognition, amplification over private TCRs or whether the thymus after birth still preserves the ability to produce public Vδ2^+^ TCRs.

TCR-sequencing data is high-dimensional data. The CDR3 sequences are typically composed of 10-30 diverse amino acids and factors such as V(D)J recombination, frequency, and MHC restriction need to be considered in the analysis of this. Recently, different computational tools were developed to discover TCR clusters based on the sequence patterns (15–17). For example, TCRdist3 is an open-source python package which transforms TCR repertoires into biochemically informed distance metrics based on the similarity of the TCR amino acid sequences, especially on the CDR3 sequence regions. The calculated distance metrics enabled clustering or meta-clonotype analysis to be carried out on the TCR sequences (18, 19). However, MHC restriction of αβ TCRs and lack of HLA genotyping data for most of the available data impeded these tools from being applied to public TCR datasets on a larger scale. In contrast, the MHC-unrestricted nature of γδ TCR makes it possible to apply TCRdist3 on γδ TCR repertoires across a large number of individuals.

To investigate the heterogeneity and ontogeny of public Vδ2^+^ TCRs, we determined the publicity of TCRs from Vδ2^+^ TCR repertoires of 89 individuals from cord blood (CB), infant peripheral blood, and adult peripheral blood. Vδ2^+^ CDR3 amino acid (CDR3aa) sequences were embedded into the distance matrix by TCRdist3 and visualized by Uniform Manifold Approximation and Projection (UMAP). We found that both the J-usage and length together defined the heterogeneity of Vδ2^+^ CDR3aa sequences. Both germline-encoded and age-dependent features were preserved among public Vδ2^+^ TCRs, indicating that they are produced in the fetal and adult thymus. Interestingly, we additionally revealed a higher expansion status of public Vδ2^+^ TCRs than private Vδ2^+^ TCRs.

## Results

### Public Vδ2^+^ clones prevail in all age groups

**Figure 1:**
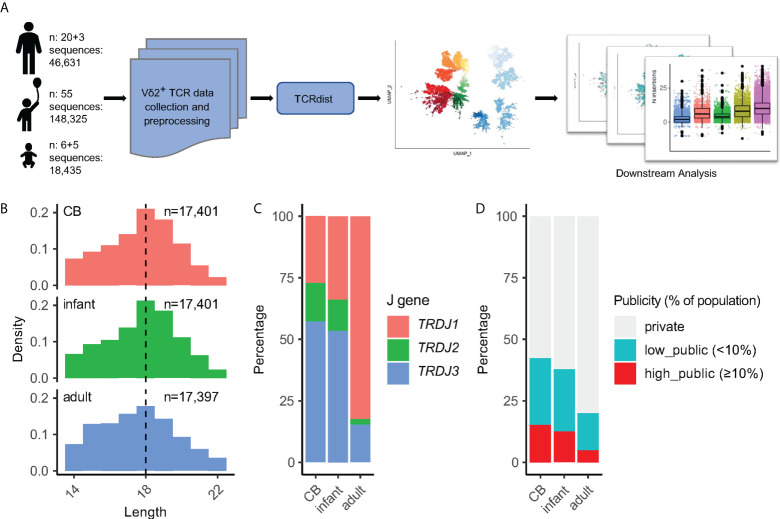
Experimental design and data pre-processing. **(A)** Illustration of the data analysis workflow. Our datasets were collected from 11 CB, 55 infants, 23 adults; among them, datasets from five CB and three adults were unpublished data. **(B)** Sequence length distribution of 52,199 CDR3aa sequences from CB, infant, and adult groups after down-sampling and pre-processing. The numbers indicate the number of CDR3aa sequences after down-sampling. Dashed lines indicate the median value of CDR3 length. **(C)** J gene usage among different age groups. **(D)** CDR3aa publicity composition among age groups. The publicity of a CDR3aa sequence is defined by the proportion of individuals that share this sequence.

To investigate the occurrence of public Vδ2^+^ clones, we collected TCR repertoires containing 213,391 Vδ2^+^ CDR3aa sequences from 11 cord blood (CB), 55 infant peripheral blood, and 23 adult peripheral blood samples. Eighty-one samples were collected from our published studies (4, 13, 20, 21), and eight of these samples (five CB and three adult) were included from an unpublished databank to increase further the sample size (**Figure 1A** and **Table S1**). The lengths of CDR3s ranged from 4 to 39 amino acids with a median of 18 amino acids (**Figure S1A**). The *TRDJ3* segment dominated in CB samples and rapidly decreased after birth. Similarly, 15.9% of the *TRDV2* rearranged with *TRDJ2* in CB, but this number decreased to around 2.1% in adults. In contrast, the *TRDJ1* segment increased to a large majority in adult samples compared to the small frequency that was found in CB. The proportions of the *TRDJ4* segment were marginal in all three groups (**Figure S1B**). “Public” Vδ2^+^ TCR clones were defined by the proportion of individuals sharing the same CDR3aa sequence Private TCR CD3R regions were found in only one individual. As well as this low and high TCR’s appeared in less than or equal to 10% of individuals respectively. In CB samples, 26.8% of TCR sequences were low public and 15.1% were high. Interestingly, although the publicity of adult Vδ2^+^ TCRs significantly decreased,14.5% of low public and 4.6% of high public TCRs were still found on average (**Figure S1C**).

Before applying the TCRdist3 tool to Vδ2^+^ TCR repertoires, data pre-processing and down-sampling were performed (**Figure 1A**). To reduce the noise caused by rare sequences, we only selected CDR3aa sequences with a length between 14 to 22 amino acids, and all *TRDJ4* rearrangements were also excluded (**Figures S1A**, **1B, C**). Subsequently, this led to 52,199 CDR3aa sequences being obtained after down-sampling. This data cleansing and down-sampling method did not significantly affect the J-usage and publicity of post-procession TCRs in this study (**Figures 1C, D**).

### Highly diverse Vδ2^+^ TCRs cluster according to CDR3aa length and *TRDJ* segment usage

**Figure 2:**
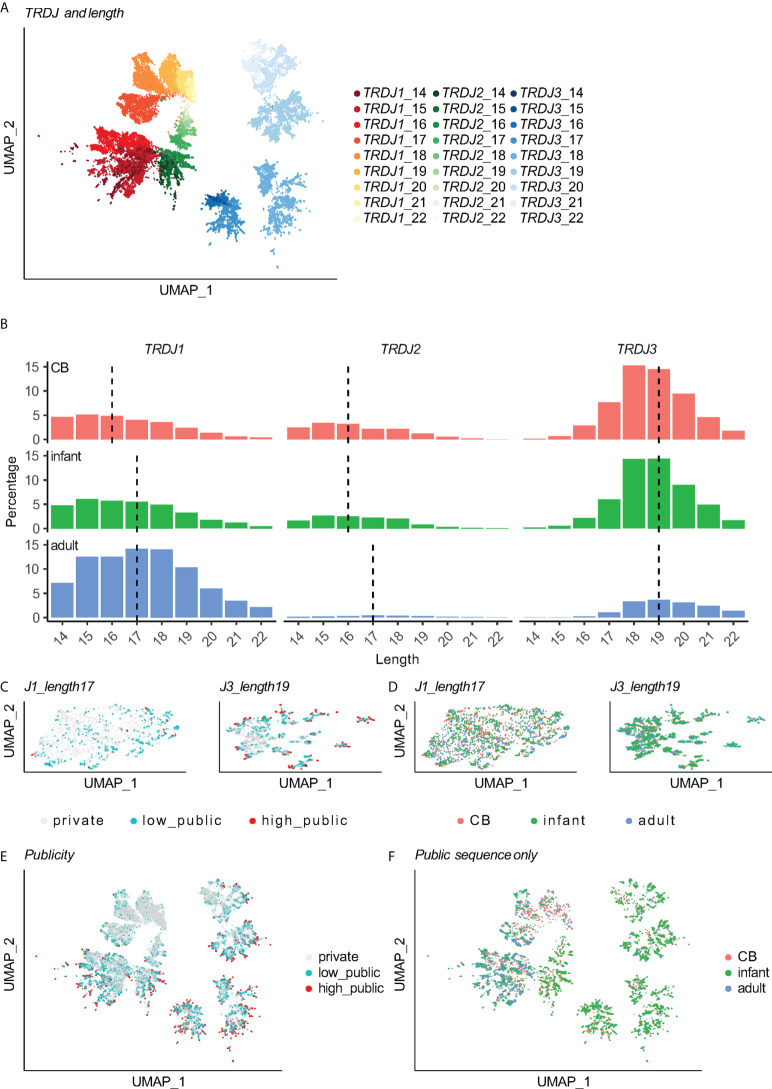
The heterogeneity of Vδ2 TCRs is determined by CDR3 lengths and *TRDJ* segments. **(A)** Each point stands for a Vδ2^+^ CDR3aa sequence. UMAP for 52,199 Vδ2^+^ CDR3 (same data as in **Figure 1B**) colored by the combination of J gene usage and CDR3aa length. **(B)** CDR3aa length distribution with different J segments and age groups. The dashed line indicates the median length. **(C)** UMAP of length = 17 *TRDJ1* Vδ2^+^ CDR3aa sequences (left), UMAP of length = 19 *TRDJ3* Vδ2^+^ CDR3aa sequences (right) colored by sequence publicity. **(D)** The same UMAPs in **(C)** are colored by age group. **(E)** Same UMAP in **(A)** colored by the publicity label of the sequence. **(F)** UMAP in **(A)** after filtering out private TCRs, colored by age group.

The distance between every two TCRs was calculated based on CDR3aa sequences by the TCRdist3 which generated a distance matrix (18, 19) Following this a UMAP was generated to allow data embedding and visualization (**Figures 1A**, **2A**). At first sight, Vδ2^+^ TCRs were clearly stratified on the UMAP by both the J-usage and CDR3aa length (**Figures S2A, B**). The J-usage skewed from *TRDJ3-* and *TRDJ2*-dominant in the CB group to *TRDJ1*-dominant in the adult group (**Figures 2B**, **S2C**). Longer CDR3s on the other hand were more frequently found in *TRDJ1* and *TRDJ2* adult group. The CDR3aa length distribution between the age groups did however remain similar (**Figures 2B**, **S2C, D**). Infant-derived Vδ2^+^ TCRs showed intermediate features between CB and adult TCRs in terms of both J-usage and CDR3aa length (**Figures 2B**, **S2C, D**).

Adjacent to this, in order to test if other factors contributed to the heterogeneity of Vδ2^+^ TCRs, we selected the TCRs with the most prevalent lengths for the *TRDJ1* (length 17aa) and *TRDJ3* (length 19aa) regions for a more in-depth re-analysis. This showed that publicity (**Figure 2C**) and age groups (**Figure 2D**) were not distinguishable on the re-analyzed UMAP. More evidently, after restricting to the same J-usage and length, the CDR3aa sequence logomap showed almost identical motifs between the different publicity and age groups (**Figures S2E, F**). This suggests that the heterogeneity of Vδ2^+^ TCRs is primarily determined by a combination of *TRDJ* usage and CDR3aa length.

### Public Vδ2^+^ repertoire preservers both germline and age-related characteristics

In previous studies by Ravens et al. and Papadopoulou et al., public Vδ2^+^ TCRs were described as germline-encoded CDR3 with either no or few N-insertions and short CDR3 lengths (12, 13). In our dataset, publicity was also reversely associated with the number of N-insertions and length of CDR3aa (**Figures S3A,B**). Interestingly, public Vδ2^+^ TCRs previously have shown age-dependent wave-like dynamics: enriching in fetal blood, then decreasing in cord blood before rising again in 5 to10-week-old infants and then finally dropping in adulthood (12, 13). This then therefore led us to determine whether or not the public clones generated in different time windows would also show similar age-dependent features. Indeed, although public Vδ2^+^ TCRs were enriched in TCR clusters with shorter lengths, they still demonstrated to preserve the J-usage and length-determined heterogeneity as private Vδ2^+^ TCRs also displayed (**Figures 2E, F**).

**Figure 3:**
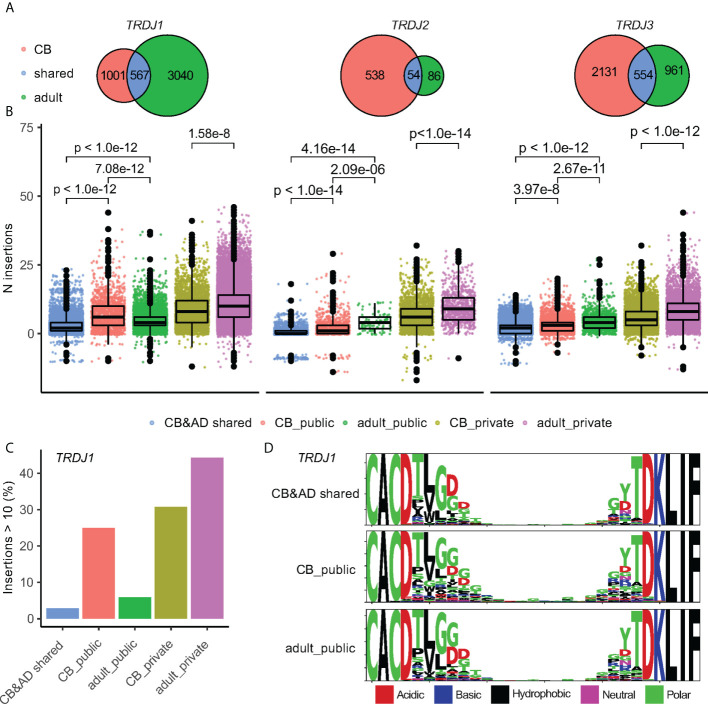
Patterns of public clones alter between age groups. **(A)** Venn plots show the overlap of public Vδ2^+^ clones between CB and adult groups. The sizes of ellipses correlate to number of unique clones. **(B)** N-insertion of Vδ2^+^ TCRs in corresponding groups. Each point stands for a CDR3 nucleotide sequence. The distribution is summarized by box plot; the three horizontal lines of the box-whisker plot represent the higher quartile, median, and lower quartile, respectively. Games-Howell test was used for P-value calculation. Vδ2^+^ TCRs were grouped by J-usage and the publicity between CB and adult. **(C)** barplot shows the ratio of sequences with N insertions ≥ 10 in publicity groups with *TRDJ1* gene usage. **(D)** Logomap for sequences with public *TRDJ1* gene usage.

Following this, to investigate how public Vδ2^+^ TCRs’ features changed during development, we took advantage of the whole dataset before down-sampling. Overlapping of all unique public CDR3aa clones for different age groups showed that only a minor portion of clones were shared between the CB and adult groups (CB&AD shared) (1,175 out of 4,641 in CB and 1,175 out of 5,262 in adult). In contrast, both CB and adult groups largely shared their public Vδ2^+^ repertoire with the infant group (4,428 out of 4,641 in CB and 4,258 out of 5,262 in adult) (**Figure S3C**). From combining the transitional features of infant TCRs in the J-usage and length, we considered that age-related differences of public Vδ2^+^ TCRs mainly exist between CB and adult groups (**Figure 3A**), while a transitional infant group shared the commonalities from both sides. As TdT activity increases along with human development, we hypothesized that adult-derived TCRs would have more N-insertions than CB-derived ones. Indeed, the private Vδ2^+^ TCRs from the adult group had the most N-insertions and longest CDR3aa length, whereas the CB&AD shared group Vδ2^+^ TCRs had the fewest N-insertions (**Figures 3B**, **S3D**). The N-insertions of adult-derived *TRDJ2* and *TRDJ3* public Vδ2^+^ TCRs were slightly more than that of CB-derived public TCRs (**Figure 3B**). Intriguingly, for *TRDJ1*, we observed more TCRs with higher N-insertions in the CB public group than in the adult public group (**Figure 3B**). Here 25.0% of CB-derived public Vδ2^+^ *TRDJ1* TCRs had more than 10 N-insertions. Whereas for adult-derived and CB&AD shared public clones, the number was merely 5.91% and 2.91%, respectively (**Figure 3C**). Finally, although the CB-derived public Vδ2^+^ *TRDJ1* TCRs had more residues in the high-variable region, the motifs of the three groups were similar, i.e. polar amino acids were mainly used (**Figure 3D**).

### Synonymous codons in CDR3 nucleotide sequences result from different TRDD-gene usages and N-insertions that contribute to the generation of public Vd2^+^ CDR3

**Figure 4:**
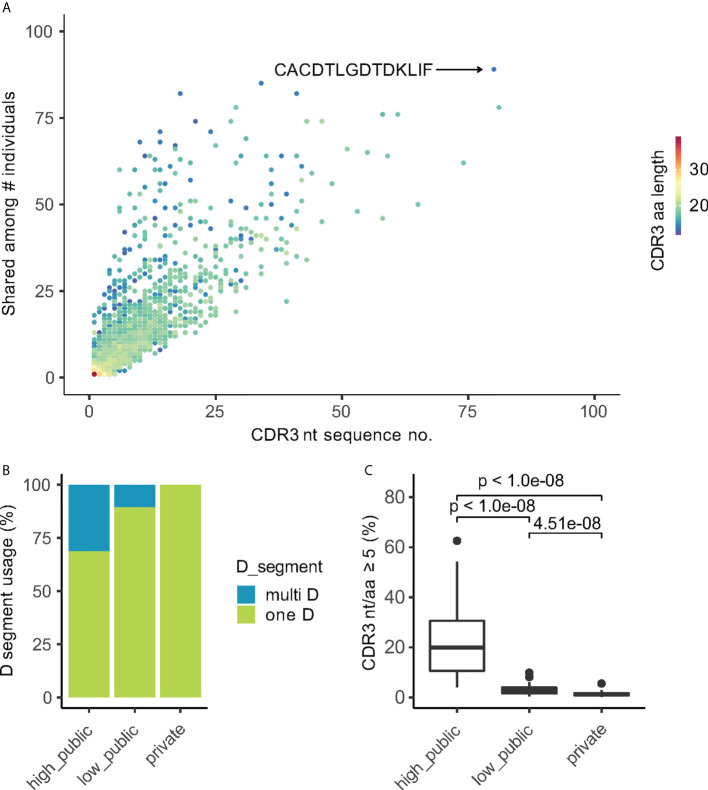
Public CDR3aa clones have more corresponding CDR3nt transcripts. **(A)** Scatter plot of the publicity of CDR3aa (number of individuals sharing the CDR3aa) vs the number of corresponding CDR3nt sequences. Each point indicates one unique CDR3aa sequence colored by the CDR3aa length. The most public CDR3aa sequence are indicated on the plot. **(B)** TRDD-segment usage of CDR3 sequences in different publicity groups. **(C)** Box plot shows the ratio of CDR3aa sequences translated from 5 or more different nucleotide transcripts in each individual. Games-Howell *Post-Hoc* Test was used to test the mean difference between groups. Adjusted P-values are shown between groups.

Since the generation of public Vδ2^+^ clones did not entirely result from simple germline rearrangements without N-insertions (**Figures 3B, C**), we explored in more detail how the public CDR3aa sequences were rearranged. The publicity of CDR3aa sequence positively correlated with the number of its corresponding unique encoding transcripts (**Figure 4A**). The same CDR3aa sequences could be generated by the exceedingly high numbers of different CDR3 nucleotide (CDR3nt) sequences. For example, the public CDR3aa sequence ‘CACDTLGDTDKLIF’ (2) was detected in 76 different individuals as well as also being transcribed from 80 different transcripts (**Figure 4A**). Additionally, public Vδ2^+^ CDR3aa sequences were more likely to have a variable *TRDD*-segment usage. 31.3% and 10.6% of ‘high public’ and ‘low public’ CDR3’s, respectively, could be rearranged from more than one *TRDD-*segment, whereas a much lower frequency of only 0.16% was observed in private CDR3’s (**Figure 4B**). More surprisingly, public CDR3aa sequences could be generated from multiple CDR3nt sequences even within one individual. For example, in donor SA62, the public CDR3 “CACDTLGDTDKLIF” could be produced by eight different CDR3nt transcripts, either rearranged with *TRDD3* and 0 – 1 N-insertion, *TRDD2* with 2 N-insertions, or 9 N-insertions without *TRDD* segment (**Table 1**). 19.8% (median value, ranging from 4% to 62.6%) of high public Vδ2^+^ CDR3 in each individual were generated by at least five unique transcripts. In contrast, the number of private CDR3s was much lower at 1.28% (median value, ranging from 0.26% to 5.56%) (**Figure 4C**).

**Table 1:** CDR3aa “CACDTLGDTDKLIF” corresponding CDR3nt sequences for individual SA62.

CDR3nt sequence	*TRDD*	*TRDJ*	N-insertion
TGTGCCTGTGACAC **C** CT **A** GG **A** GA **C** ACCGATAAACTCATCTTT	*TRDD2*	*TRDJ1*	2
TGTGCCTGTGACAC **C** CTGGGGGATACCGATAAACTCATCTTT	*TRDD3*	*TRDJ1*	0
TGTGCCTGTGACAC **A** CTGGGGGATACCGATAAACTCATCTTT	*TRDD3*	*TRDJ1*	0
TGTGCCTGTGACAC **G** CTGGGGGATACCGATAAACTCATCTTT	*TRDD3*	*TRDJ1*	1
TGTGCCTGTGACAC **T** CTGGGGGATACCGATAAACTCATCTTT	*TRDD3*	*TRDJ1*	1
TGTGCCTGTGACAC **T** CTGGGGGATAC **T** GATAAACTCATCTTT	*TRDD3*	*TRDJ1*	1
TGTGCCTGTGACAC **A** CTGGGGGA **C** ACCGATAAACTCATCTTT	*TRDD3*	*TRDJ1*	0
TGTGCCTGTGACAC **C** CT **A** GG **C** GATACCGATAAACTCATCTTT	.	*TRDJ1*	9

Under scores indicate variable nucleotide residues.

### The publicity of Vδ2^+^ clones positively associated with expansion status

**Figure 5:**
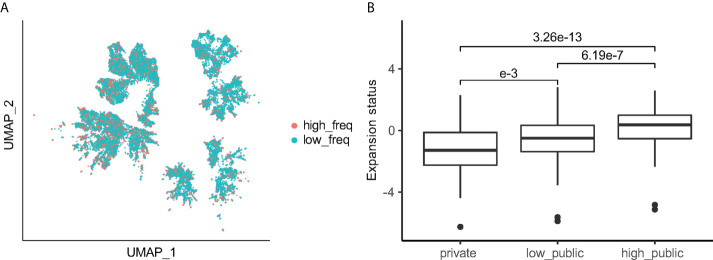
Public clones have a greater expansion status compared to private clones. **(A)** UMAP of Vδ2^+^ TCR colored by high-freq/low-freq category. **(B)** Expansion status score for each publicity group. Games-Howell *Post-Hoc* Test was used to test the mean difference between groups. Adjusted P-values are shown between groups.

To determine whether the publicity of Vδ2^+^ TCRs correlated to the expansion ability, we assigned the top 25% of most expanded TCRs in each sample as high frequency (high-freq) TCRs and then labelled the remaining as low frequency (low-freq) TCR’s (**Figure S4A**). The high-freq and low-freq TCRs were not distinguishable on the UMAP (**Figure 5A**). In order to understand which groups of Vδ2^+^ TCRs are more likely to be high-freq TCR’s, we calculated the “expansion status score” based on high-freq to low-freq TCRs (Methods section). For a group of TCRs in one individual, the expansion status is calculated by dividing the number of high-freq TCRs in the group by the number of low-freq TCRs followed by a log-transformation. Hence, the higher the expansion status score, the more high-freq TCR’s in that group. An expansion status score of > 0 means the group has more high-freq TCRs than low-freq ones. Interestingly, the median expansion status score of “high public” TCRs was 0.37, and that of the “low public” TCR’s remained significantly higher than the private TCR values (-0.50 vs -1.28, median value) (**Figure 5B**). We further examined the expansion status score for TCRs with different J-usages, and similar results were observed (**Figure S4B**). Given that the publicity is reversely associated with CDR3 length (**Figure 4A**), expansion status could also be associated with CDR3 length. However, CDR3aa lengths only demonstrated to have a minimal impact on expansion status, and the median expansion status scores of all lengths and *TRDJ*s remained below 0 (**Figures S4C, D**).

## Discussion

In this study, we applied TCRdist3 to systematically investigate the Vδ2^+^ TCR repertoire and revealed that Vδ2^+^ TCRs retain a high heterogeneity that is primarily determined by the J-usage and CDR3aa length. It was observed that public Vδ2^+^ TCRs were as diverse as private TCRs. In previous studies, TCR’s with high publicity or shared between cord blood (CB) and adult age groups were characterized to show only a few or no N-insertions and shorter CDR3length (6, 13). Unexpectedly, our study also demonstrated that the *TRDJ1* of public (but not of private) γδ TCRs in CB displayed a relatively high number of N-insertions and longer CDR3 lengths. Moreover, it was additionally revealed that, compared to private Vδ2^+^ CDR3aa sequences, the public Vδ2^+^ CDR3aa sequences were prone to be generated from multiple CDR3nt transcripts even within one individual. Thus, it could be concluded that germline-like rearrangement and synonymous codons used by CDR3nt sequences contribute to the generation of public CDR3aa. Finally, public Vδ2^+^ TCRs displayed a higher expansion status than private Vδ2^+^ TCRs.

By using TCRdist3 and various other tools for investigating CDR3 motif or amino acid properties ‘clustering’ of TCR’s can be carried out. This strategy was particularly useful in linking αβ TCR sequences to antigen-specificity based on similarity (18, 22–24). In contrast to highly rearranged αβ TCRs, which have the ability to recognize any possible antigen, most of the rearranged Vγ9Vδ2^+^ TCRs are instead generated from relatively fixed options and are thought to uniformly recognize phosphoantigens (2, 7). Complex TCR repertoire data can be extracted to generate a single UMAP by applying the TCRdist3 method to conveniently analyze the heterogeneity of γδ TCR’s. Hence, it is useful when investigating the shift of the Vδ2^+^ TCR repertoire under different physiological and pathological conditions. For example, in our study, the repertoire shift from CB-derived to adult-derived repertoire was notably highlighted. Moreover, from this, it would be interesting to see if TCRdist3 could be applied to the more adaptive Vδ1^+^ or Vδ3^+^ γδ TCRs to possibly determine their function and antigen-specificity.

Vδ2^+^ TCRs derived after birth displayed more N-insertions and longer CDR3 length than those from CB, considering the increasing TdT activity. However, in contrast to this, the public clones among CB-derived *TRDJ1* Vδ2^+^ TCRs showed more N-insertions and longer CDR3 than their adult public TCR counterparts. This property was not seen among public Vδ2^+^ TCRs with other J-usage meaning it is difficult to fully explain and understand this complex feature as yet. One possibility for this could be that it may associate with the intrathymic differentiation of Vγ9Vδ2^+^ T effectors. Mouse and human innate γδ T effectors are committed in waves within the fetal thymus, and have shown to acquire phenotypes that are closely related with certain TCR usages (3, 4, 6, 25). While the development of human γδ T cells is not fully elucidated, it could be hypothesized that a number of underappreciated Vγ9Vδ2^+^ T effectors develop later in the fetus when the TdT becomes much more active. These specialized effector cells do not remain in peripheral blood after birth. By comparing specific γδ T cells from mice relevant information can be obtained. Mouse Vγ6^+^ and Vγ4^+^ IL-17-producing γδ T cells are a rare population of cells which reside in mucosal tissues like the skin or lungs (26, 27). These specialized cells exclusively develop at embryonic days of E15 to E18 after gestation in the fetal thymus where they will then home to specific tissues (28). Thus, there is only a narrow window in which these cells can easily be observed whilst they travel within the circulation. This therefore means that the existence of previously unknown tissue-resident γδ T cell populations which are generated shortly after birth cannot be excluded.

We demonstrated that the publicity of Vδ2^+^ TCRs positively associates with a higher expansion status. This remains in line with previous studies which also suggest that higher abundance was found on high public clones (12, 13). One of the most debatable questions regarding public Vδ2^+^ TCRs continues to determine if the generation and expansion of public Vδ2^+^ TCRs are driven by interactions with BTN2A1 and BTN3A1 butyrophilin molecules. It is also yet to be discovered if the recognition of specific antigens may additionally alter the expansion process within these Vδ2^+^ TCRs. Although the CDR3 is essential for recognition, previous studies failed to find evidence that the CDR3 of Vδ9Vδ2^+^ TCRs specifically recognize phosphoantigens (7–10). We cannot exclude the possibility that even the family of Vγ9Vδ2^+^ TCRs recognizes antigens in an “adaptive-like” way *via* the CDR3 until a complete structure of interacting Vγ9Vδ2^+^ TCR, phosphoantigen, and butyrophilins BTN2A1 and BTN3A1 is revealed. However, based on the current understanding of Vγ9Vδ2^+^ T cells, it is unlikely that public or expanded Vδ2^+^ TCR clones result from antigen-specific clonal expansion. First of all, previous *ex vivo* experiments suggested that phosphoantigen stimulation induced both polyclonal and unbiased expansion of Vδ9Vδ2^+^ T cells (6, 20). Moreover, in our study, by calculating the geometric distance between Vδ2^+^ CDR3 based on sequence patterns, it was found that there is no significant difference between public and private Vδ2^+^ CDR3 patterns or between high-freq and low-freq Vδ2^+^ CDR3s. The results suggest that the binding between Vδ2^+^ CDR3 and phosphoantigen-activated butyrophilins BTN2A1 and BTN3A1 does not favor specific CDR3 variants or motifs.

Taking this all into account it can be determined why public Vδ2^+^ TCRs appear to have a survival advantage? Based on the rearrangement bias and development ontogeny, various speculations can be made as follows: 1). There is a rearrangement bias. where the publicity of Vδ2^+^ TCR CDR3aa positively associates with the number of corresponding CDR3nt sequences. Therefore, the γδ T cells with a public Vδ2^+^ TCR may have multiple sources from different TCR rearrangements, resulting in a higher copy number. 2). Most public Vδ2^+^ TCRs, especially those shared between many individuals, are rearranged early in life and persist into adulthood (4, 12–14). They may simply have more time to accumulate. A similar situation was observed in human αβ T cells, where it was found that T cells carrying public αβ TCRs were generated before birth and then continued to maintain high abundances for a long time throughout adulthood (29).

One of the major limitations to this current study was that it was only viable to investigate the Vδ2^+^ chains, meaning information on the corresponding pairing of Vγ9 chains was lost. Within our study it was also difficult to prove or disapprove the possibility that public Vγ9Vδ2^+^ TCRs may interact with antigens in a different way compared to antigen interaction by private TCRs. However, recent advancements in single-cell TCR sequencing do make it possible to sequence paired γδ TCR and relate it to phenotypes of other cells (4). From this it can be expected that more such data will soon become available. Another limitation to this study was the fact that undersampling could possibly impair accuracy. As the library protocol only enabled a survey of up to tens of thousands of γδ T cells from a portion of a PBMC sample, this underrepresented the huge vast number of γδ T cells that are actually living within our body. This undersampling may make it difficult to accurately identify moderately expanded clones. However, considering the relatively low diversity of Vδ2^+^ TCRs, undersampling may compromise some details, but the major findings are unlikely to be greatly affected.

The TCRdist3 method has proven to be a very useful tool for analyzing human αβ T cells, and the software is able to support γδ TCR analysis (19). However, as mentioned above, the αβ TCRs have a much higher heterogeneity than Vδ2^+^ TCRs provided by the V(D)J rearrangement. Thus, detecting the different patterns between αβ TCRs is considerably easier. In our case, the TCRdist3 detected the heterogeneity of Vδ2^+^ TCRs generated by length and J-usage, but not by publicity or age. Our sequence pattern analysis also failed to find heterogeneity between public and private TCRs. Furthermore, the existing possibility that more subtle and essential substitutions hiding in public Vδ2^+^ TCRs cannot be excluded. Currently, methods for TCR clustering are all based on the CDR3aa sequences, which is sufficient to study antigen-specificity. However, the ontogeny of TCRs can be better determined if CDR3nt sequences are included to provide crucial information about VDJ rearrangement and N-insertions.

Our study established that TCR sequence analysis tools such as the TCRdist3 are very useful for investigating the γδ TCR repertoire. By using TCRdist3 and downstream analysis, it could be demonstrated that public Vδ2^+^ TCRs are a heterogeneous population with both germline and age-related features that confer expansion advantages over private TCRs. Given that expressing γδ TCRs on αβ T cells is a promising immunotherapy strategy against tumors (30, 31), those “more successful” public Vγ9Vδ2 TCR might improve the performance of immunotherapy using Vγ9Vδ2^+^ T cell clones or engineered αβ T cells carrying Vγ9Vδ2 TCR.

## Materials and methods

### Human sample isolation and preparation

Data from 8 healthy donors in this study were newly generated. Blood samples from adult donors (n = 3) and cord blood (CB) donors (n = 5) were collected at Hannover Medical School (Hannover, Germany) after written informed consent. This study was performed in accordance with the Declaration of Helsinki and approved by the institutional ethics review board at Hannover Medical School under study numbers 1303-2012 (CB individuals) and 7901-2018 (healthy adult individuals). PBMCs and CBMCs were purified from the blood samples by Ficoll-Paque density gradient media separation. These cells were then stored at -80°C in 90% fetal bovine serum and 10% DMSO freezing medium before use.

### Vγ9Vδ2^+^ T cells sorting

Fluorescence-activated cell sorting (FACS) was performed using the FACS Aria Fusion flow cytometer (BD, USA). PBMC and CBMC were incubated with 5% Fc-receptor block before staining. The following antibodies were used: anti-CD3 (clone REA613; Miltenyi Biotec), anti-CD3 (clone SK7; BD Bioscience), anti-γδ TCR (clone 11F2, BD Bioscience or Miltenyi Biotec), anti-Vγ9 (clone IMMU 360; Beckman Coulter), anti-Vδ2 (clone 123R3; Miltenyi Biotec).

### Vδ2^+^ TCR library construction and sequencing

All the newly generated data was sequenced and pre-processed in the same way as other published data used in this study (4, 13, 20, 21). Briefly, RNA was extracted from sorted DAPI^─^CD3^+^γδ^+^Vγ9^+^Vδ2^+^ cells from PBMC or CMBC by an RNAeasy Micro Kit (Qiagen). Reverse transcription was carried out with Superscript III reverse transcriptase (Invitrogen) and oligo(dT) primers. As previously described (21), δ chains was amplified *via TRDV2* specific primers hTRDV2: ATTGCAAAGAACCTGGCTGT and hTRDC: GACAAAAACGGATGGTTTGG. The PCR program was set as follows: 1). 95°C for 3 min; 2). 95°C, 63°C, and 72°C for 30s each, for 5 cycles; 3). 95°C for 30s, 72°C for 35s, for 20–25 cycles; 4). 72°C for 4 min.

The amplified cDNA library with Illumina P5 and P7 adaptor was sequenced by Illumina Miseq using 500 cycles of paired-end sequencing.

### Raw sequencing data alignment and annotation

Raw reads alignment annotation was performed with MiXCR software v.2.1.12 to international immunogenetics information system (IMGT) reference (32). Unproductive TCRs were filtered out. Annotated TCRs were further counted and summarized by VDJtools (33).

### Data integration and processing

VDJtools output files from all the 89 individuals from the published and newly generated datasets were merged together. Since some datasets comprised entire TCRδ repertoires (13, 21), non-Vδ2 TCRs were filtered out. Numbers of N-insertions were calculated *via* VDJtools output as following: For TCRs rearranged with a *TRDD* segment: N-insertion = (Jstart – Dend – 1) + (Dstart – Vend - 1); For TCRs without D-usage: N-insertion = Jstart – Vend – 1.

Vend, Dstart, Dend, Jstart are the start/end position of V, D, J segments on CDR3nt sequence.

Publicity of TCRs were defined based on the CDR3aa sequence by whether a sequence is shared among a certain percentage of the population. “private” CDR3aa is defined as CDR3aa that only appears in only one individual, “high public” TCRs are shared among at least 10% of the population, i.e. shared among 9 or more individuals in our study, the remaining TCRs are defined as “low public”, i.e. shared by at least 2 individuals to 10% of the population.

### TCR distance calculation and UMAP embedding

Vδ2^+^ CDR3aa sequences with a length from 14 to 22 aa were preselected and downsampled for TCR distance calculation. CDR3s rearranged with *TRDJ4* segment were excluded. For each age group, the numbers of CDR3s were randomly down-sampled to 17,398 - 17,401 sequences. TCR distances were computed according to the protocol of TCRdist3 (34). Briefly, CDR3aa sequence, V-usage, and J-usage were then included as input for the TCRdist3 in the Python 3.8 environment. CDR1, CDR2, and CDR2.5 sequences were reconstructed from the V-usage. After alignment, penalties were given to each mismatch between two TCRs according to the BLOSUM62 substitution matrix. Finally, distance was calculated as the weighted sum of penalties across all CDRs. The TCR distance matrix was further embedded into latent spaces by UMAP.

### Calculation of expansion potential

In each individual, CDR3aa sequences were ranked by the frequencies from high to low. The top 25% of CDR3s were assigned as “high frequency” TCRs, and the rest were labelled as “low frequency” TCRs. (**Figure S4A**). The expansion status score is calculated for a pre-defined group of TCRs within an individual (*i.e.* the high public *TRDJ1* Vδ2^+^ TCR in the donor CB2) as follows:


$$Expansion\;status\;score=\ln(\frac{n_{\text{highfreq}}+1}{n_{\text{lowfreq}}+1})$$

n*_highfreq_* and n*_lowfreq_* are the number of high-freq and low-freq CDR3aa sequences in the group.

### Sequence alignment and logomap

Sequences of selected groups were aligned using Clustal Omega (35–37), logomap was generated from the aligned sequences using Logomaker (38).

### Statistics

Statistical analyses were performed under R v4.1.2. The statistical methods are described in the figure legends, in all cases, considering the sample size, variance and number of comparisons. Either a one-way ANOVA or a Tukey’s HSD test after a one-way ANOVA or Games-Howell *Post-Hoc* Test was used and *P*-values were then calculated.

## Data availability statement

The previously unpublished raw data presented in this study are deposited in the GEO repository, accession number GSE213280. All codes and processed data are available from Github repository https://github.com/isihh-uke/gdTCR_analysis.git.

## Ethics statement

The studies involving human participants were reviewed and approved by Institutional ethics review board at Hannover Medical School. Written informed consent to participate in this study was provided by the participants’ legal guardian/next of kin.

## Author contributions

LD and LT conducted and interpreted bioinformatics analysis. AH and SR organized and performed TCR sequencing. LD, IP, and LT designed the study and wrote the manuscript. All authors contributed to the article and approved the submitted version.

## Funding

This work was supported by the Deutsche Forschungsgemeinschaft (DFG; German Research Foundation) Research Unit FOR 2799 to SR and IP (Project ID 395236335) and under Germany’s Excellence Strategy, EXC 2155 “RESIST,” (Project ID 390874280) to IP and SR.

## Acknowledgments

We thank Ms. Alexandra Morse for proofreading this manuscript. We would like to acknowledge the assistance of the Cell Sorting Core Facility at the Hannover Medical School supported in part by Deutsche Forschungsgemeinschaft and the IT department at Center for Molecular Neurobiology Hamburg, University Medical Center Hamburg-Eppendorf.

## Conflict of interest

The authors declare that the research was conducted in the absence of any commercial or financial relationships that could be construed as a potential conflict of interest.

## Publisher’s note

All claims expressed in this article are solely those of the authors and do not necessarily represent those of their affiliated organizations, or those of the publisher, the editors and the reviewers. Any product that may be evaluated in this article, or claim that may be made by its manufacturer, is not guaranteed or endorsed by the publisher.

## Supplementary material

Supplementary Figure 1 CDR3 delta chain sequence distribution among different age groups. **(A)** Sequence length distribution of 213,391 CDR3aa sequences from 89 individuals, including 11 CB, 55 infants, and 23 adults. The numbers on the plot indicate the number of CDR3s in each group. Black dashed lines indicate the median value of CDR3 length. Grey dash lines indicate the range of length of CDR3aa sequences that are used for TCRdist3 computation. **(B)** J gene composition among different age groups. **(C)** CDR3 publicity composition among age groups. The publicity of a CDR3aa sequence is defined by the proportion of individuals that share this sequence.

Supplementary Figure 2 The heterogeneity of Vδ2 TCRs is determined by CDR3 lengths and *TRDJ* segments. UMAP in colored by **(A)** J-usage and **(B)** CDR3aa length. **(C)** Contour plot visualization on the density distribution of TCRs on the UMAP, split by age groups. **(D)** Boxplot of CDR3aa sequence length for different age groups by different J gene usage. Adjusted *P-*values calculated from Tukey’s HSD test after one-way ANOVA are shown between groups. **(E,F)** Logomap for length = 17 *TRDJ1* (left) length = 19 *TRDJ3* (right) Vδ2^+^ TCRs in **(E)** different age groups and **(F)** different publicity groups.

Supplementary Figure 3 Sequence length and N insertion exploration for CDR3 sequences in different publicity groups. **(A)** N-insertion of Vδ2^+^ TCRs. Each point stands for a CDR3 nucleotide sequence. The distribution is summarized by a box plot. (**B**) CDR3 aa length of Vδ2^+^ TCRs with different J gene usages and different publicity groups. (**A,B**) P-values calculated from one-way ANOVA are shown. (**C**) Venn plots show the overlap of public Vδ2^+^ clones between CB, infant, and adult groups. The sizes of ellipses correlate to the number of unique clones. (**D**) CDR3 aa length of Vδ2^+^ TCRs from CB and adults with different J gene usages and publicity sharing groups. Each point stands for a CDR3aa sequence.

Supplementary Figure 4 Public clones have greater expansion status compared to private clones. **(A)** The frequency distribution for the CDR3aa sequences in different individuals, from left to right, shows three representative individuals. The low/high frequency label was defined within each individual using the corresponding 75th percentile number of the frequency as a threshold. **(B)** Expansion potential for each publicity group according to different J gene usage. Games-Howell *Post-Hoc* Test was used to test the mean difference between groups. Adjusted P-values are shown between groups. **(C)** Expansion potential for *TRDJ1* sequences with different length. **(D)** Expansion potential for *TRDJ3* sequences with different length.

## References

[1] Market E Papavasiliou FN. V(D)J recombination and the evolution of the adaptive immune system. PLoS Biol (2003) 1(1):24–7. doi: 10.1371/journal.pbio.0000016 PMC212695 14551913

[2] Papadopoulou M Sanchez Sanchez G Vermijlen D. Innate and adaptive Γδ T cells: How, when, and why. Immunol Rev (2020) 298(1):99–116. doi: 10.1111/imr.12926 33146423

[3] Fichtner AS Ravens S Prinz I. Human Γδ tcr repertoires in health and disease. Cells (2020) 9(4):800–. doi: 10.3390/cells9040800 PMC7226320 32225004

[4] Tan L Fichtner AS Bruni E Odak I Sandrock I Bubke A. A fetal wave of human type 3 effector Γδ cells with restricted tcr diversity persists into adulthood. Sci Immunol (2021) 6(58):eabf0125–eabf. doi: 10.1126/sciimmunol.abf0125 33893173

[5] Dimova T Brouwer M Gosselin F Tassignon J Leo O Donner C. Effector Vgamma9vdelta2 T cells dominate the human fetal gammadelta T-cell repertoire. Proc Natl Acad Sci USA (2015) 112(6):E556–65. doi: 10.1073/pnas.1412058112 PMC4330771 25617367

[6] Papadopoulou M Tieppo P McGovern N Gosselin F Chan JKY Goetgeluk G. Tcr sequencing reveals the distinct development of fetal and adult human Vγ9vδ2 T cells. J Immunol (2019) 203(6):1468–79. doi: 10.4049/jimmunol.1900592 31413106

[7] Karunakaran MM Willcox CR Salim M Paletta D Fichtner AS Noll A. Butyrophilin-2a1 directly binds germline-encoded regions of the Vγ9vδ2 tcr and is essential for phosphoantigen sensing. Immunity (2020) 52(3):487–98.e6. doi: 10.1016/j.immuni.2020.02.014 32155411 PMC7083227

[8] Rigau M Ostrouska S Fulford TS Johnson DN Woods K Ruan Z. Butyrophilin 2a1 is essential for phosphoantigen reactivity by gammadelta T cells. Science (2020) 367(6478). doi: 10.1126/science.aay5516 31919129

[9] Yang Y Li L Yuan L Zhou X Duan J Xiao H. A structural change in butyrophilin upon phosphoantigen binding underlies phosphoantigen-mediated Vγ9vδ2 t cell activation. Immunity (2019) 50(4):1043–53.e5. doi: 10.1016/j.immuni.2019.02.016 30902636

[10] Yuan L Ma X Yang Y Li X Ma W Yang H. Phosphoantigens are molecular glues that promote butyrophilin 3a1/2a1 association leading to Vγ9vδ2 T cell activation. BioRxiv (2022). doi: 10.1101/2022.01.02.474068

[11] Tieppo P Papadopoulou M Gatti D McGovern N Chan JKY Gosselin F. The human fetal thymus generates invariant effector gammadelta T cells. J Exp Med (2020) 217(3). doi: 10.1084/jem.20190580 PMC7062527 31816633

[12] Papadopoulou M Dimova T Shey M Briel L Veldtsman H Khomba N. Fetal public Vgamma9vdelta2 T cells expand and gain potent cytotoxic functions early after birth. Proc Natl Acad Sci USA (2020) 117(31):18638–48. doi: 10.1073/pnas.1922595117 PMC7414170 32665435

[13] Ravens S Fichtner AS Willers M Torkornoo D Pirr S Schöning J. Microbial exposure drives polyclonal expansion of innate Γδ T cells immediately after birth. Proc Natl Acad Sci USA (2020) 117(31):18649–60. doi: 10.3389/fimmu.2018.00510 PMC7414158 32690687

[14] Holtmeier W Witthoft T Hennemann A Winter HS Kagnoff MF. The tcr-delta repertoire in human intestine undergoes characteristic changes during fetal to adult development. J Immunol (1997) 158(12):5632–41. 9190911

[15] De Neuter N Bittremieux W Beirnaert C Cuypers B Mrzic A Moris P. On the feasibility of mining Cd8+ T cell receptor patterns underlying immunogenic peptide recognition. Immunogenetics (2018) 70(3):159–68. doi: 10.1007/s00251-017-1023-5 28779185

[16] Huang H Wang C Rubelt F Scriba TJ Davis MM. Analyzing the mycobacterium tuberculosis immune response by T-cell receptor clustering with Gliph2 and genome-wide antigen screening. Nat Biotechnol (2020) 38(10):1194–202. doi: 10.1038/s41587-020-0505-4 32341563 PMC7541396

[17] Zhang H Zhan X Li B. Giana allows computationally-efficient tcr clustering and multi-disease repertoire classification by isometric transformation. Nat Commun (2021) 12(1):1–11. doi: 10.1038/s41467-021-25006-7 34349111 PMC8339063

[18] Dash P Fiore-Gartland AJ Hertz T Wang GC Sharma S Souquette A. Quantifiable predictive features define epitope-specific T cell receptor repertoires. Nature (2017) 547(7661):89–93. doi: 10.1038/nature22383 28636592 PMC5616171

[19] Mayer-Blackwell K Schattgen S Cohen-Lavi L Crawford JC Souquette A Gaevert JA. Tcr meta-clonotypes for biomarker discovery with Tcrdist3 enabled identification of public, hla-restricted clusters of sars-Cov-2 tcrs. eLife (2021) 10:1–32. doi: 10.7554/eLife.68605 PMC8631793 34845983

[20] Fichtner AS Bubke A Rampoldi F Wilharm A Tan L Steinbrück L. Tcr repertoire analysis reveals phosphoantigen-induced polyclonal proliferation of Vγ9vδ2 T cells in neonates and adults. J Leukocyte Biol (2020) 107(6):1023–32. doi: 10.1002/JLB.1MA0120-427RR 32064671

[21] Ravens S Schultze-Florey C Raha S Sandrock I Drenker M Oberdörfer L. Human Γδ T cells are quickly reconstituted after stem-cell transplantation and show adaptive clonal expansion in response to viral infection. Nat Immunol (2017) 18(4):393–401. doi: 10.1038/ni.3686 28218745

[22] DeWitt WS Yu KKQ Wilburn DB Sherwood A Vignali M Day CL. A diverse lipid antigen–specific tcr repertoire is clonally expanded during active tuberculosis. J Immunol (2018) 201(3):888–96. doi: 10.4049/jimmunol.1800186 PMC6057832 29914888

[23] Glanville J Huang H Nau A Hatton O Wagar LE Rubelt F. Identifying specificity groups in the T cell receptor repertoire. Nature (2017) 547(7661):94–8. doi: 10.1038/nature22976 PMC5794212 28636589

[24] Meysman P De Neuter N Gielis S Bui Thi D Ogunjimi B Laukens K. On the viability of unsupervised T-cell receptor sequence clustering for epitope preference. Bioinformatics (2019) 35(9):1461–8. doi: 10.1093/bioinformatics/bty821 30247624

[25] Prinz I Silva-Santos B Pennington DJ. Functional development of Γδ T cells. Eur J Immunol (2013) 43(8):1988–94. doi: 10.1002/eji.201343759 23928962

[26] Jin C Lagoudas GK Zhao C Bullman S Bhutkar A Hu B. Commensal microbiota promote lung cancer development *Via* Γδ T cells. Cell (2019) 176(5):998–1013.e16. doi: 10.1016/j.cell.2018.12.040 30712876 PMC6691977

[27] Tan L Sandrock I Odak I Aizenbud Y Wilharm A Barros-Martins J. Single-cell transcriptomics identifies the adaptation of Scart1+ Vγ6+ T cells to skin residency as activated effector cells. Cell Rep (2019) 27(12):3657–71. doi: 10.1016/j.celrep.2019.05.064 31216482

[28] Haas JD Ravens S Düber S Sandrock I Oberdörfer L Kashani E. Development of interleukin-17-Producing Γδ T cells is restricted to a functional embryonic wave. Immunity (2012) 37(1):48–59. doi: 10.1016/j.immuni.2012.06.003 22770884

[29] Pogorelyy MV Elhanati Y Marcou Q Sycheva AL Komech EA Nazarov VI. Persisting fetal clonotypes influence the structure and overlap of adult human T cell receptor repertoires. PLoS Comput Biol (2017) 13(7):e1005572. doi: 10.1371/journal.pcbi.1005572 28683116 PMC5500008

[30] Conejo-Garcia JR Innamarato P. Γδ T cells share the spotlight in cancer. Nat Cancer (2022) 3(6):657–8. doi: 10.1038/s43018-022-00396-9 35764741

[31] Marcu-Malina V Heijhuurs S Van Buuren M Hartkamp L Strand S Sebestyen Z. Redirecting αβt cells against cancer cells by transfer of a broadly tumor-reactive Γδt-cell receptor. Blood (2011) 118(1):50–9. doi: 10.1182/blood-2010-12-325993 21566093

[32] Bolotin DA Poslavsky S Mitrophanov I Shugay M Mamedov IZ Putintseva EV. Mixcr: Software for comprehensive adaptive immunity profiling. Nat Methods (2015) 12(5):380–1. doi: 10.1038/nmeth.3364 25924071

[33] Shugay M Bagaev DV Turchaninova MA Bolotin DA Britanova OV Putintseva EV. Vdjtools: Unifying post-analysis of T cell receptor repertoires. PLoS Comput Biol (2015) 11(11):e1004503–e. doi: 10.1371/journal.pcbi.1004503 PMC4659587 26606115

[34] Mayer-Blackwell K Schattgen S Cohen-Lavi L Crawford JC Souquette A Gaevert JA. Tcr Meta-Clonotypes for Biomarker Discovery with Tcrdist3 Enabled Identification of Public, Hla-Restricted Clusters of Sars-Cov-2 Tcrs. Elife (2021) 10:1–32. doi: 10.7554/eLife.68605 PMC8631793 34845983

[35] Sievers F Higgins DG. Clustal omega for making accurate alignments of many protein sequences. Protein Sci (2018) 27(1):135–45. doi: 10.1002/pro.3290 PMC5734385 28884485

[36] Sievers F Wilm A Dineen D Gibson TJ Karplus K Li W. Fast, scalable generation of high-quality protein multiple sequence alignments using clustal omega. Mol Syst Biol (2011) 7(1):539. doi: 10.1038/msb.2011.75 21988835 PMC3261699

[37] Sievers F Barton GJ Higgins DG. Multiple sequence alignments. In: Baxevanis AD Wishart DS, editors. Bioinformatics. Wiley (2020). p. 227–50. GDB4.

[38] Tareen A Kinney JB. Logomaker: Beautiful sequence logos in Python. Bioinformatics (2020) 36(7):2272–4. doi: 10.1093/bioinformatics/btz921 PMC7141850 31821414

