# Corrigendum: Systematic pattern analyses of Vδ2^+^ TCRs reveal that shared “public” Vδ2^+^ γδ T cell clones are a consequence of rearrangement bias and a higher expansion status

**DOI:** 10.3389/fimmu.2022.1055103

**Published:** 2022-10-06

**Authors:** Lihua Deng, Anna Harms, Sarina Ravens, Immo Prinz, Likai Tan

**Affiliations:** ^1^ Institute of Systems Immunology, University Medical Center Hamburg-Eppendorf, Hamburg, Germany; ^2^ Institute of Immunology, Hannover Medical School, Hannover, Germany

**Keywords:** γδ TCR, Vγ9Vδ2^+^ T cells, TCR distance, TCR sequencing, *TRD* rearrangement

In the published article, the name of the proofreading editor was incorrectly spelled in the **Acknowledgements** section as “Ms. Alexandra Mores”. It should be “Ms. Alexandra Morse”.

The full correct **Acknowledgements** appears below:

“We thank Ms. Alexandra Morse for proofreading this manuscript. We would like to acknowledge the assistance of the Cell Sorting Core Facility at the Hannover Medical School supported in part by Deutsche Forschungsgemeinschaft and the IT department at Center for Molecular Neurobiology Hamburg, University Medical Center Hamburg-Eppendorf”.

The authors apologize for this error and state that this does not change the scientific conclusions of the article in any way. The original article has been updated.

## Publisher’s note

All claims expressed in this article are solely those of the authors and do not necessarily represent those of their affiliated organizations, or those of the publisher, the editors and the reviewers. Any product that may be evaluated in this article, or claim that may be made by its manufacturer, is not guaranteed or endorsed by the publisher.

