# Supplementary figures and images for: Systematic pattern analyses of Vδ2+ TCRs reveal that shared “public” Vδ2+ γδ T cell clones are a consequence of rearrangement bias and a higher expansion status
Source: Front Immunol. 2022 Sep 27;13:960920. doi: 10.3389/fimmu.2022.960920 (PMC9583675; doi:10.3389/fimmu.2022.960920)

A

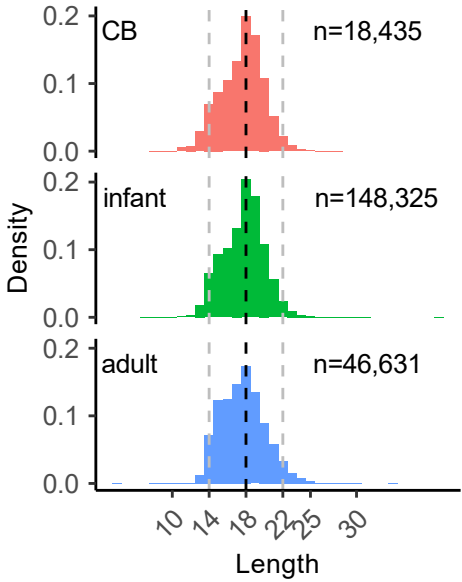

B

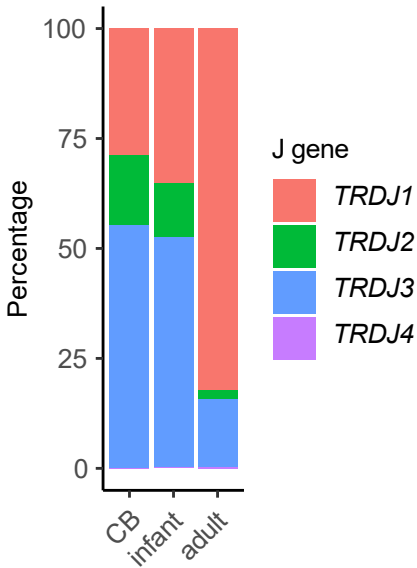

C

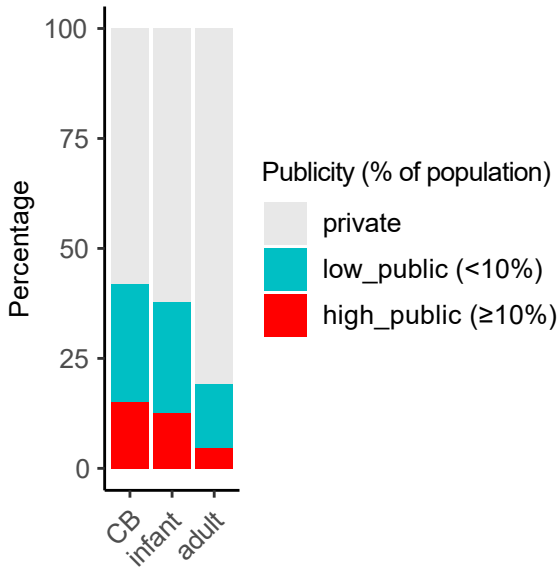

FigS2

**A** *TRDJ*

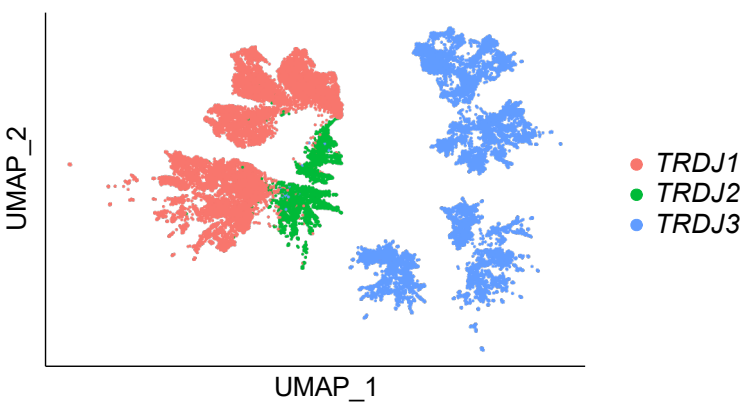

**B** *Length*

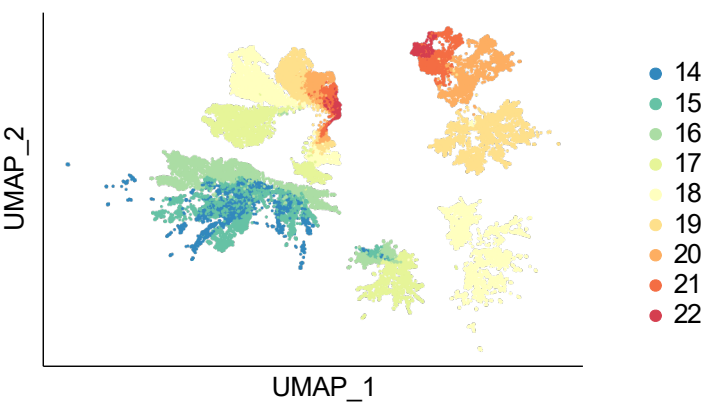

**C**

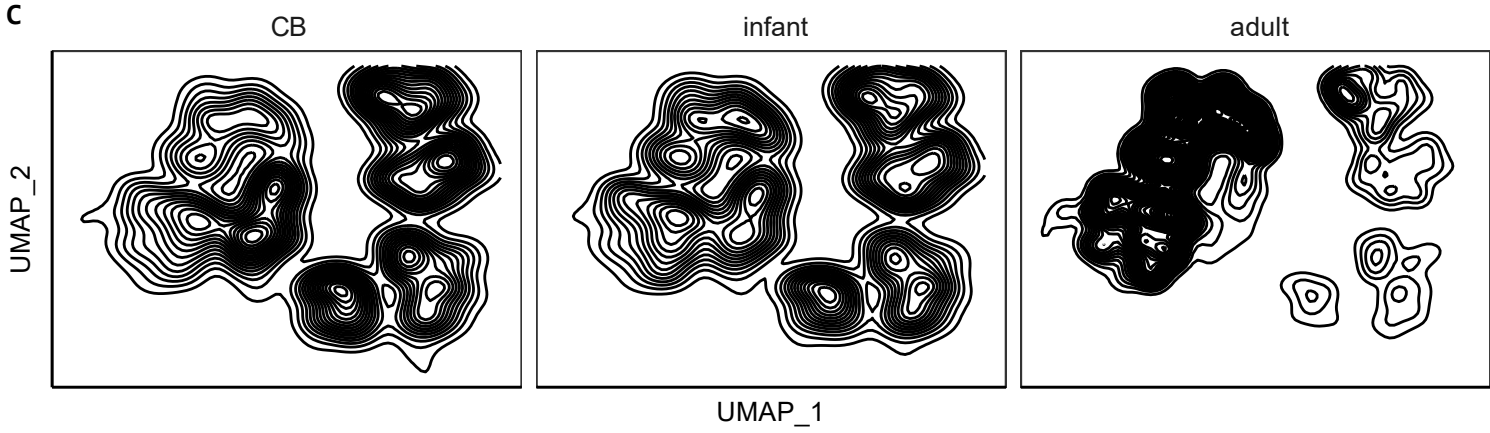

**D**

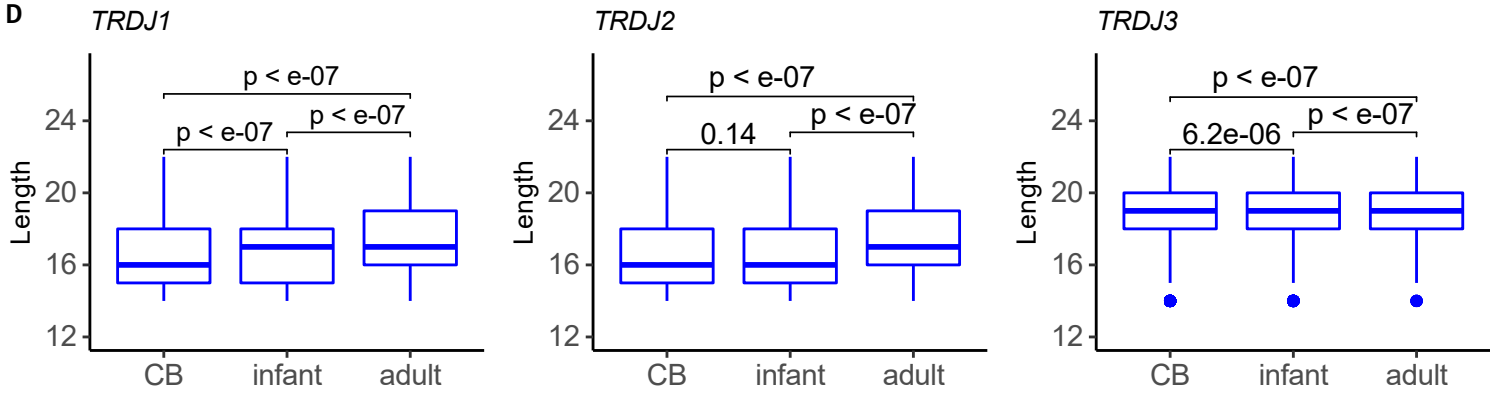

**E** *TRDJ1\_17*

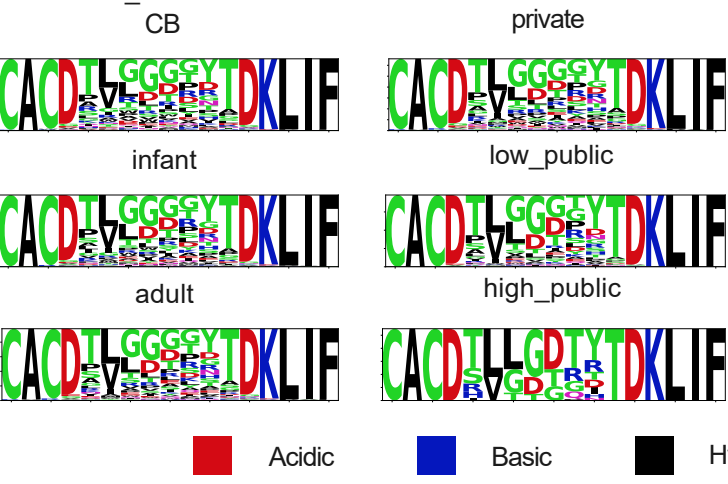

**F** *TRDJ3\_19*

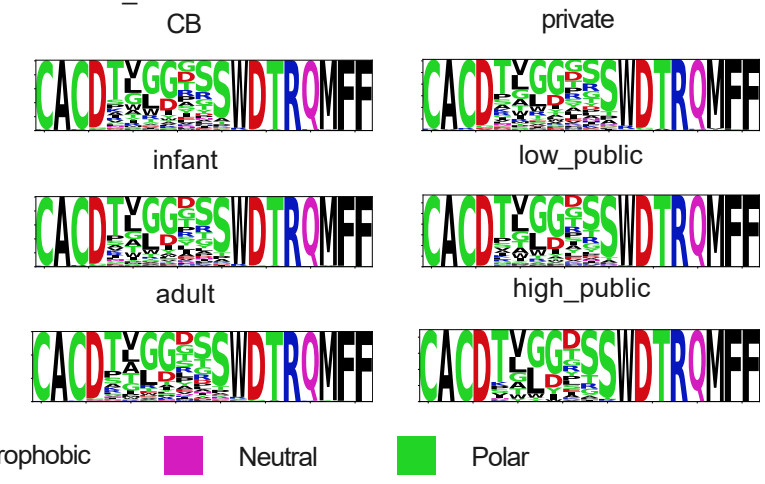

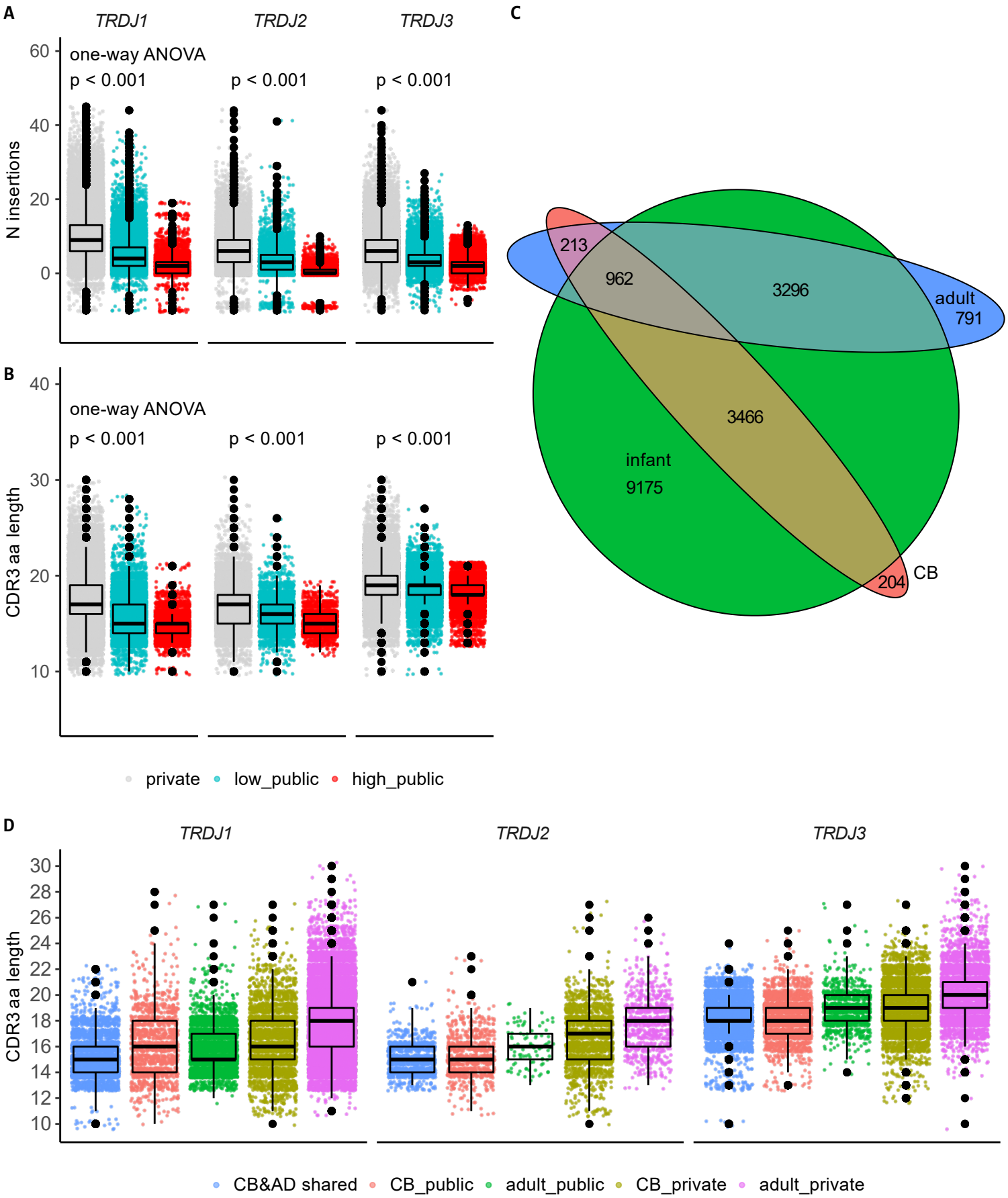

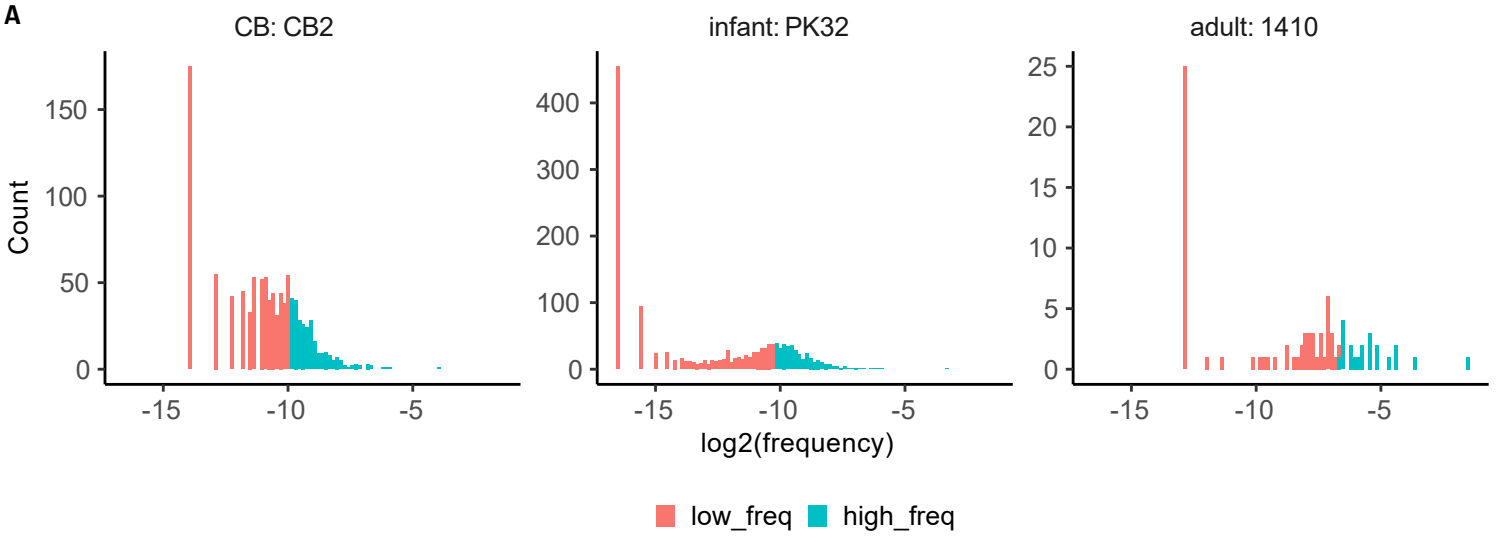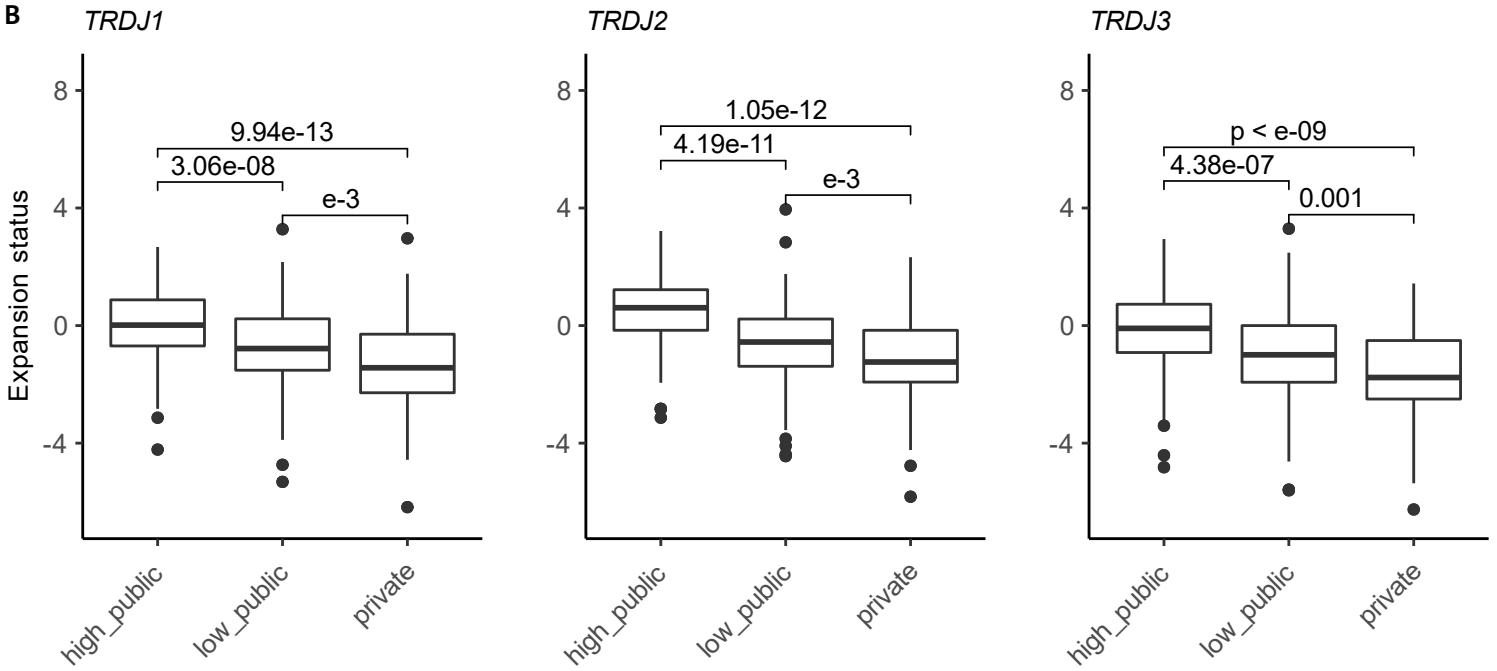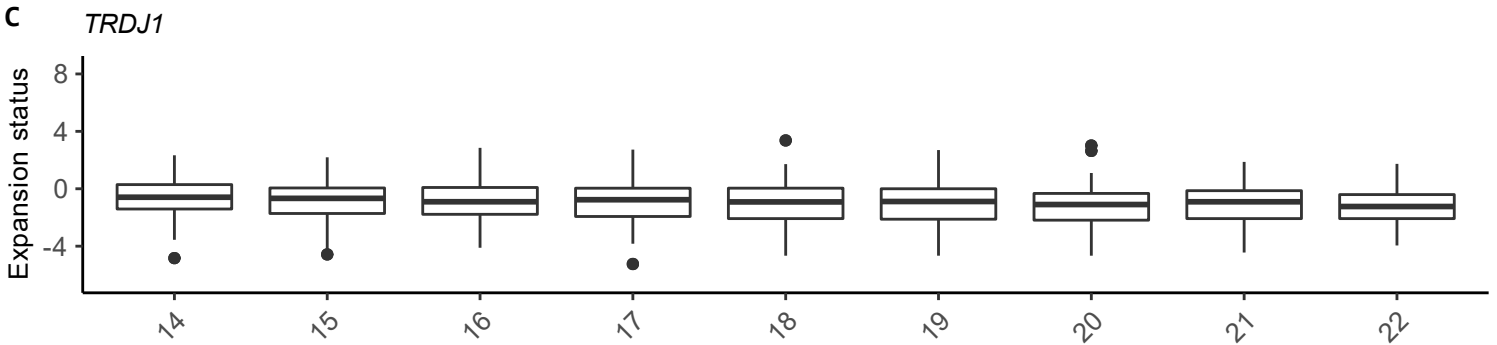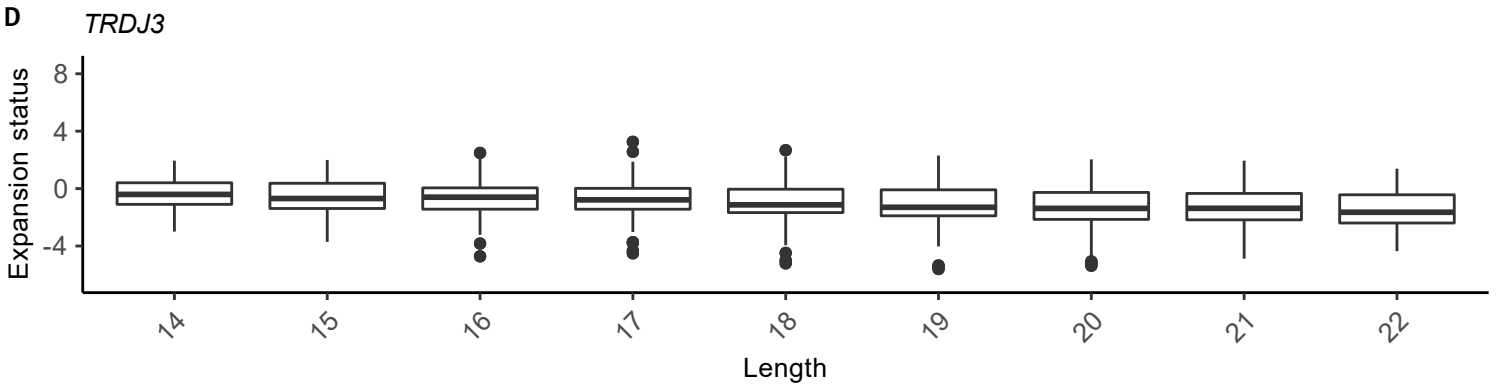

Supplement: Supplementary Figure 1 — CDR3 delta chain sequence distribution among different age groups. (A) Sequence length distribution of 213,391 CDR3aa sequences from 89 individuals, including 11 CB, 55 infants, and 23 adults. The numbers on the plot indicate the number of CDR3s in each group. Black dashed lines indicate the median value of CDR3 length. Grey dash lines indicate the range of length of CDR3aa sequences that are used for TCRdist3 computation. (B) J gene composition among different age groups. (C) CDR3 publicity composition among age groups. The publicity of a CDR3aa sequence is defined by the proportion of individuals that share this sequence. [file Image_1.pdf]
